# Supplementary material for: Parents’ knowledge and practices of child eye health care: A scoping review
Source: PLoS One. 2024 Nov 14;19(11):e0313220. doi: 10.1371/journal.pone.0313220 (PMC11563364; doi:10.1371/journal.pone.0313220)
Supplement: Attachment — Submitted filename: Response to Reviewers.pdf [file pone.0313220.s002.pdf]

Manuscript Comment and Correction

No	Comment	Correction
1	Please ensure that your manuscript meets PLOS ONE's style requirements, including those for file naming. The PLOS ONE style templates can be found at https://journals.plos.org/plosone/s/file?id=wjVg/PLOSOne_formatting_sample_main_body.pdf and https://journals.plos.org/plosone/s/file?id=ba62/PLOSOne_formatting_sample_title_authors_affiliations.pdf	Thank you for the comment. This manuscript has been amended to follow PLOS ONE guidelines.
2	Thank you for stating the following financial disclosure: "Universiti Kebangsaan Malaysia research grant (GUP-2020-086)"<br><br>Please state what role the funders took in the study. If the funders had no role, please state: "The funders had no role in study design, data collection and analysis, the decision to publish, or preparation of the manuscript." If this statement is not correct you must amend it as needed. Please include this amended Role of Funder statement in your cover letter; we will change the online submission form on your behalf.	Thank you for the comment. This statement has been amended and included in the online submission form and cover letter.
3	Thank you for stating the following in the Acknowledgments Section of your manuscript:<br>"This work was supported by Universiti Kebangsaan Malaysia research grant (GUP-2020-086)"<br><br>We note that you have provided funding information that is not currently declared in your Funding Statement. However, **funding information should not appear in the Acknowledgments section** or other areas of your manuscript. We will only publish funding information present in the Funding Statement section of the online submission form.<br><br>Please remove any funding-related text from the manuscript and let us know how you would like to update your Funding Statement. Currently, your Funding Statement reads	Thank you for the comment. This statement has been removed from the manuscript.

	as follows:<br>"Universiti Kebangsaan Malaysia research grant (GUP-2020-086)"<br><br>Please include your amended statements within your cover letter; we will change the online submission form on your behalf.	
**4**	We note that your Data Availability Statement is currently as follows: "All relevant data are within the manuscript."<br><br>Please confirm at this time whether or not your submission contains all the raw data required to replicate the results of your study. Authors must share the "minimal data set" for their submission. PLOS defines the minimal data set to consist of the data required to replicate all study findings reported in the article, as well as related metadata and methods (https://journals.plos.org/plosone/s/data-availability#loc-minimal-data-set-definition).<br><br>For example, authors should submit the following data:<br><br>- The values behind the means, standard deviations and other measures reported;<br>- The values used to build graphs;<br>- The points extracted from images for analysis.<br><br>Authors do not need to submit their entire data set if only a portion of the data was used in the reported study.<br><br>If your submission does not contain these data, please either upload them as Supporting Information files or deposit them to a stable, public repository and provide us with the relevant URLs, DOIs, or accession numbers. For a list of recommended repositories, please see https://journals.plos.org/plosone/s/recommended-repositories.<br><br>If there are ethical or legal restrictions on sharing a de-identified data set, please explain them in detail (e.g., data contain potentially sensitive information, data are owned by a third-party organization, etc.) and who has imposed them (e.g., an ethics committee). Please also provide contact information for a data access committee, ethics committee,	Thank you for the comment. The statement in the online submission form has been changed to the minimal data set are within the manuscript.

	or other institutional body to which data requests may be sent. If data are owned by a third party, please indicate how others may request data access.	
**5**	Please ensure that you refer to Figure 1 in your text as, if accepted, production will need this reference to link the reader to the figure.	Thank you for the comment. Figure 1 has been added to the text.
**6**	Please include captions for your Supporting Information files at the end of your manuscript, and update any in-text citations to match accordingly. Please see our Supporting Information guidelines for more information: http://journals.plos.org/plosone/s/supporting-information.	Not applicable.
**7**	Please update your submission to use the PLOS LaTeX template. The template and more information on our requirements for LaTeX submissions can be found at http://journals.plos.org/plosone/s/latex.	Thank you for the comment. This manuscript has been amended to follow the PLOS LaTeX template.
**Additional comments**		
8	Your revised manuscript should include a more detailed methodology specifying the McMaster Critical Appraisal Tool, its scoring system, the reasons for the exclusion of a large number of studies identified and why only eleven were included in the final review.	Thank you for highlighting this issue. Detailed methods for McMaster Critical Appraisal Tools and its scoring system have been added in the **methodology:** selecting study section (lines 132-147).<br><br>Based on the PRISMA Scr guideline, during the titles and abstracts screening, only articles that are relevant to the research question are included. A large number of articles were excluded from this review because the title and abstract were not relevant. Explanation for this has been added in the **result** section (lines 174-181).

9	The results should detail specific questions or questionnaires that were included in the studies, and the Tables (specifically Table 5) should be modified.	Thank you for the comment. In this review, seven studies used questionnaires, one study used in-depth interviews and three studies used mixed methods (questionnaire, interview, and focus group discussion). All the studies used different questionnaires, each of which had been piloted and validated. Among the questions asked regarding parents' knowledge in the studies were eye care, education of the visually impaired, refractive error, and eye disease.<br><br>Details regarding the mode of data collection used in the studies to obtain information from parents in each study have been added in Table 5 column 2.
10	In addition, when reporting means, it would be advisable to also report the range of the scores.	Thank you for the comment. The range of the McMaster score in this review was 6-11. This statement has been added in the methodological quality section (lines 217-219).
-11	The discussion should include the association between knowledge and practice patterns, and the implications of the parent's knowledge to visual dysfunctions and their developmental, occupational, and economic consequences.	Thank you for the comment. The association between knowledge and practice is discussed in lines 324-326 and the implications of parents' knowledge have been included in lines 374-376.
12	It would be advisable to have your final draft edited by an English speaker to make sure the timing and the grammar are correct.	Thank you for the comment. This manuscript has been sent for proofreading as suggested.

Reviewer 1

No	Comment	Correction
**Abstract**		
1	There is no data regarding the populations that were included in the articles that were included in the review.	Thank you for the comment. This review included all studies that were performed on parents with children aged from birth to 18 years old.
2	How did the information was obtained from the parents – by questionnaire? By a valid one? Is it the same questionnaire in all studies that were included?	Thank you for the comment.<br><br>In this review, seven studies used questionnaires, one study used in-depth interviews and three studies used mixed methods (questionnaire, interview, and focus group discussion). All the studies used different questionnaires, each of which had been piloted and validated.<br><br>Details regarding the mode of data collection used in the studies to obtain information from parents in each study have been added in Table 5 column 2.
3	Age range of children that was addressed in the parent's knowledge assessment is missing.	Thank you for the comment. In this review, all studies performed on parents with children (aged from birth to 18 years old) were included.
4	What keywords were used for the systematic literature review? Any excluded studies and why?	Thank you for the comment. The keywords (lines 31-32) used for the literature search and the reason for excluded studies (lines 37-40) have been added to the abstract.

**Introduction**		
5	Line 58: it is better to detail which vision impairments could cause the mentioned complications.	Thank you for the comment. The statement was quoted from the WHO, which did not specify the type of visual impairment. The definition of visual impairment in that article is "Vision impairment occurs when an eye condition affects the visual system and its vision functions."
6	Please explain the mechanism in which vision problems and amblyopia development could cause have consequences later on developmental, educational, and occupational consequences.	Thank you for the comment. The statement regarding the mechanism of vision problems that leads to developmental, educational, and occupational consequences has been added in the introduction (lines 66-68).
7	Line 67: stigma is not the main cause for low knowledge on children's eye problems and parents who do not seek for eye examination on time to their child. Please find additional main obstacles for parents who do not adhere to vision exams in the following study: https://pubmed.ncbi.nlm.nih.gov/35970195/	Thank you for highlighting this issue. True, stigma and belief are not the only reasons for low knowledge of children's eye problems among parents. This paragraph has been amended accordingly (lines 73-82).
8	Please give examples of intervention programs that succeed in lowering amblyopia rates and it's consequences following good parental knowledge and adherence to recommendations. Such as: USPSTF. The following articles may be helpful: https://aapos.org/members/vision-screening-guidelines, https://pubmed.ncbi.nlm.nih.gov/28873168/ , https://pubmed.ncbi.nlm.nih.gov/31514739/ , https://pubmed.ncbi.nlm.nih.gov/25647611/	Thank you for the comment. An example of the intervention program that improved adherence to eye care has been added in the introduction (lines 77-80)

Method		
9	Please specify if the McMaster Critical Appraisal Tool is software or what kind of tool.	Thank you for the comment. McMaster Critical Appraisal Tools is a form that has a set of questions designed to assess the validity and reliability of the research study. In this review, this form was completed for all selected studies.<br><br>Detailed methods for McMaster Critical Appraisal Tools and its scoring system have been added in the selecting study section (lines 136-143).
10	There is no mention of this tool and the number of studies that were excluded by this tool on the diagram of Figure 1. Please specify.	Thank you for the comment. McMaster Critical Appraisal Tools have been described in the selecting study section (lines 132-143).<br><br>McMaster Critical Appraisal Tools was conducted on the 11 studies that have been selected. Based on the evaluation, all the 11 studies were accepted. This was not included in Figure 1, it is not part of the PRISM Scr.
11	Please specify which questionnaires were used to assess parent's knowledge. An example of such a questionnaire and its validity process can be found in the following study, which also should be mentioned in the discussion: https://pubmed.ncbi.nlm.nih.gov/37620666/	Thank you for the comment.<br><br>In this review, seven studies used questionnaires, one study used in-depth interviews and three studies used mixed methods (questionnaire, interview, and focus group discussion). All the studies used different questionnaires to assess parents' knowledge, each of which had been piloted and validated.

		Details regarding the mode of data collection used in each study to obtain information from parents have been added in Table 5 column 2.
12	Figure 1: please detail regarding the reasons for the massive exclusion of papers. Was it done by the McMaster tool or by the author?	Thank you for the comment.<br><br>Figure 1 is the flow diagram for PRISMA ScR. PRISMA ScR was used for the methodology and reporting of this scoping review.<br><br>Based on the PRISMA Scr guideline, during the titles and abstracts screening, only articles that are relevant to the research question are included. A large number of articles were excluded from this review because the title and abstract were not relevant. Explanation for this has been added in the **result** section (lines 174-181).<br><br>The exclusion of studies was done by the author based on PRISMA ScR guidelines. The McMaster Critical Appraisal Tools was used to evaluate the selected studies (after PRISMA ScR).

**Result**		
13	Lines 119-121: it is mentioned that this tool provides a dichotomous score (yes/no), but on lines 185-188 and table 4 it gives a score of 6-11. Please explain.	Thank you for the comment. McMaster Critical Appraisal Tools used dichotomous scores which yes= 1, and no= 0. The range of the McMaster score in this review was 6-11. This statement has been added in the methodological quality section (lines 217-219).
14	Tables 4-5: very good tables. Please detail the title of table 5.	Thank you for the comment. The title for Table 5 has been revised to accurately reflect its contents.
**Discussion**		
15	Please detail the main findings in the first paragraph.	Thank you for the comment. The main findings have been discussed in a separate paragraph Knowledge and Practice
16	Are there any other examples from other fields on health literacy and its effect on health status.	Thank you for the comment. The statement regarding health literacy and its effect on health status has been added (lines 289-295)
17	Please add additional reasons for parents who do not adhere to vision exams: https://pubmed.ncbi.nlm.nih.gov/35970195/	Thank you for the comment. The additional reason for parents who do not adhere to vision exam has been added (lines 309-314)
18	Line 320: no need for the "LIMITATIONS" title. It is part of the discussion.	Thank you for the comment. The word “limitation” has been changed to “Study Limitation” following the latest Plos One manuscript format.
19	Please explain why so many studies were excluded from the analysis. Could be that so many studies are with low quality regarding assessment of parent's awareness?	Thank you for the comment.<br><br>Based on the PRISMA Scr guideline, during the titles and abstracts screening, only articles that are relevant to the research question are included. A large number of articles were excluded from this review because the title and

		abstract were not relevant. Explanation for this has been added in the **result** section (lines 174-181).

Reviewer 2

No	Comment	Correction
1	Table 5 contains percentages regarding awareness and practice. However, it is not clear what those percentages refer to. For example, in paper 7, does 71.4% refer to the percentage of parents who “know about the benefit of wearing spectacles”? The authors must explain how they got the percentages in the methods and in the table legend.	Thank you for the comment. The percentages in Table 5 refer to the percentage of parents. This has been amended for better understanding. The percentages were taken directly from the selected studies.
2	In the paragraph describing parents’’ knowledge – how is the category “knowledgeable” defined? Are the authors using the percentages in table 5? If so, what is the cut-off? 50%? Same question regarding the categories “aware”, “did not have knowledge” and “misconceptions.”	Thank you for the comment. The statement regarding knowledgable has been amended. Yes, the result discussed was based on the percentages in Table 5.
3	The same questions apply to parents’ practice (lines 219-230). How were the categories “practice positive attitude” and “negative practice” defined? Was this based on the percentages in table 5? If so, when there is more than one statement, on which is it based?	Thank you for the comment. The percentage in Table 5 refers to parents. The sentence has been amended accordingly.
4	In addition, it will make Table 5 easier to understand if the authors color-coded the awareness and practice columns.	Thank you for the comment. The knowledge and practice were separated by a column. The colour was used to separate the studies and findings to avoid confusion.
5	The authors should put an asterisk on studies carried out in developed countries.	Thank you for the comment. The developed country in Table 5 was marked with an asterisk.
6	Did the authors find an association between “knowledge” and “practice”?	Thank you for the comment. Yes, the association between knowledge and practice were discussed in the discussion section (lines 324-326).
7	The English in the introduction could be improved.	Thank you for the comment. This manuscript has been sent for proofreading as suggested.
8	Line 118 has the word (name?) pearling in parentheses. What is this referring to?	Thank you for the comment. The explanation for Pearling has been added in the methodology section (lines 130-133)

9	Results line 151-2. Please state why three were excluded. Due to which criteria?	Thank you for the comment. There are four excluded studies and this has been amended in Figure 1 and in the text (lines 177-179). The four studies were excluded because they did not meet the research questions and objectives after full-text review. The explanation for the exclusion has been added in the **result** section (lines 177-179).
10	Line 188 – only one numeral after the decimal place.	Thank you for the comment. This has been amended as suggested.
11	Table 4 – what do the percentages refer to?	Thank you for the comment. The percentage in Table 4 represents the total score in percent. This has been amended in Table 4 and explained in the methodology: stage 3: selecting a study section (lines 140-143).
12	In the discussion, starting line 249, the authors write "majority of parents have knowledge about children's eye health care and common eye problem in children." This cannot be concluded from the study. The scoping review found that in majority of studies parents have knowledge. We do not know if this can be extrapolated to the majority of parents.	Thank you for the comment. This statement has been amended to accurately reflect the findings (lines 243-266).
13	It would be interesting to compare the findings from studies in developed countries to those in less developed countries.	Thank you for the comment. Regrettably, we did not compare the finding between developed and less developed countries, since there are only two studies done in the developed countries and the results are same.
