# Supplementary material for: Parents’ knowledge and practices of child eye health care: A scoping review
Source: PLoS One. 2024 Nov 14;19(11):e0313220. doi: 10.1371/journal.pone.0313220 (PMC11563364; doi:10.1371/journal.pone.0313220)
Supplement: Attachment — Submitted filename: Response to Reviewers.pdf [file pone.0313220.s003.pdf]

Response to Reviewers

No	Comment	Correction
**Additional Editor Comments**		
**1**	Some minor additional comments that will improve the manuscript: Abstract: parents have a "huge" responsibility – perhaps "paramount" is a better term?	Thank you for the comment. The term "huge" has been changed to "paramount" to better reflect the critical importance of the responsibility parents have in managing their children's eye health.
**2**	Line 57: Not sure that the word "Shockingly" is required and appropriate for a scientific report. Perhaps better rephrased. Amongst the 36 million individuals worldwide affected by vision impairment as reported by the World Health Organization (WHO), 29% are children, and this figure will rise [2].	Thank you for the comment. The word "shockingly" has been removed, and this statement has been amended as suggested.
**3**	Line 230: that parents are knowledgeable	Thank you for the comment. This statement has been amended.
**4**	Study 4 does not have percentages at all.	Thank you for the comment. Study 4 is a qualitative study that uses semi-structured interviews, therefore, percentages were not provided by authors. This has been stated in table 5.
**5**	Study 9, knowledge – "parents believe that routine eye exam is not necessary – this has no score."	Thank you for the comment. In Table 5, it has been specified that more than 57% of parents believe routine eye exams are not necessary.
**6**	The captions for Tables 4 and 5 should be modified to describe the numbers provided in parentheses and what they represent.	Thank you for the comment. The captions for Table 4 and 5 have been updated to clearly describe the number provided in parentheses and what they represent.

Reviewer 2

No	Comment	Correction
**Abstract**		
1	The manuscript is vastly improved and the authors addressed most of the critique provided by the reviewers. This includes providing more information on the methodology used.<br>However, the classifications in table 5 and interpretations, as raised by reviewer 2, remain unclear. In the methods section, lines 162-167 the authors define good and poor as follows:<br>Parents' level of knowledge and practice were categorized as either 'good' or 'poor' based on Bloom's cut-off point in line with several KAP studies [26,27]. Parents with a knowledge score of more than 60% were classified as possessing good knowledge, while those parents who had a score of less than 60% were classified as having poor knowledge. As for practice, parents scoring 80% and above were categorized as demonstrating good practice,<br>while those parents who had a score less than 80% were classified as displaying poor practice.<br>However, in table 5, this is not clear – for example – study 1 it appears that 78.2% had poor knowledge, 18.2 had good knowledge and 3.6 excellent knowledge. Meaning, the authors provided the distribution and not a category based on above or below 60%. In study 2, "parents with blind…. Have knowledge about eye problems 24.7%" meaning poor? Again in study 3, "Parents know about their child's eeye problems 89.5% and 10% do not know." This is a distribution. The next line is "parents know where to go for eye examinations 98.2%." Does this mean they have a knowledge score above 60% or 98.2% know where to go?	Thank you for your details feedback. In the revised manuscript the categorization of knowledge and practices based on Bloom's cut-off points had been deleted because it has caused confusion. The percentages reported in Table 5 represent parents and not the score of knowledge and practices. Thus, we do not used Bloom's cut-off point in Table 5. Table 5 in this revised manuscript reported the actual findings of percentages of parents from the selected studies.
2	Study 4 does not have percentages at all.	Thank you for the comment. Study 4 is a qualitative study that uses semi-structured interviews, therefore, percentages were not provided by authors. This has been stated in table 5.

3	Study 9, knowledge – “parents believe that routine eye exam is not necessary – this has no score.”	Thank you for the comment. In Table 5, it has been specified that more than 57% of parents believe routine eye exams are not necessary.
