# Supplementary material for: Parents’ knowledge and practices of child eye health care: A scoping review
Source: PLoS One. 2024 Nov 14;19(11):e0313220. doi: 10.1371/journal.pone.0313220 (PMC11563364; doi:10.1371/journal.pone.0313220)
Supplement: Attachment — Submitted filename: Response to Reviewers.pdf [file pone.0313220.s004.pdf]

Manuscript Comment and Correction

No	Comment	Correction
**Additional Editor Comments**		
**1**	The authors have sufficiently addressed the reviewers' past comments. However, there are still some minor comments to address before the paper can be accepted for publication as listed below:<br>Line 106 of this scoping review is to identify<br>WAS to identify	Thank you for the comment. This statement has been amended.
**2**	Table 1 Treatment capitol T	Thank you for the comment. This has been amended.
**3**	Following the PRISMA ScR 178 guideline, only studies relevant to the research question were included while screening titles and abstracts.<br>This sentence should be moved so it precedes the following:<br>Upon examining titles and abstracts, the majority (204) of the studies were excluded, leaving 15 studies for thorough evaluation through full-text review.<br>And this sentence is superfluous and can be deleted:<br>A significant number of studies were excluded from this review due to their lack of relevance in the title and abstract.	Thank you for your comment. The sentences have been corrected as suggested.
**4**	Line 247 were undertaken in developed countries (England and XXX) [17,20].	Thank you for the comment. This statement has been amended. The two studies were undertaken in England only. So, now the sentence reads as below:<br><br>The majority of the studies were conducted in developing countries – India [13,16], Nigeria [7,9,21], Saudi Arabia [11,15], Sudan [18], Swaziland [19] , and only two (n=2) studies were undertaken in a developed country (England) [17,20].

**5**	Table 5 asterisk: *This was a qualitative study, percentages are not available.<br>Asterisk: developed country	Thank you for the comment. In Table 5, it has been amended; asterisk is to represent developed country. A bracket has beed added to the sentence "This was a qualitative study, percentages are not available"
**6**	Line 273: practice instead of practices	Thank you for your comment. The word has been corrected as suggested.
**7**	Line 279: Overall, this review	Thank you for the comment. This statement has been amended.
**8**	Line 299: half of the parent	Thank you for the comment. This statement has been amended.
**9**	Line 301: these matters instead of such matters	Thank you for the comment. This statement has been amended.
**10**	Line 303: suggest	Thank you for your comment. The word has been corrected as suggested.
**11**	Line 304: comma after income	Thank you for the comment. The comma has been added.
**12**	Line 312: motivates instead of motivated	Thank you for your comment. The word has been corrected as suggested.
**13**	Line 314: Based on instead of according to<br>Also- some of the studies instead of the selected studies	Thank you for the comment. This statement has been amended as suggested.
**14**	Lines 324-325: necessity of seeking proper treatment options and weighing potential risks and benefits for their children's eye health care.	Thank you for the comment. This statement has been amended as suggested.
**15**	Line 332" In this review, more than half of the selected studies	Thank you for the comment. This statement has been amended as suggested.
**16**	Line 334: period instead of comma after the references [18.20]	Thank you for your comment. This has been amended.
**17**	Line 334: Five of the selected studies reported	Thank you for the comment. This statement has been amended as suggested.
**18**	Line 369: are unnecessary	Thank you for your comment. This has been amended.

**19**	Line 393: Limitations (plural)	Thank you for your comment. This has been amended.
**20**	Some studies found that parents are knowledgeable and some found stigmas and lack of knowledge. Can the authors please address if there is a relationship between the knowledge vs. stigma and lack of knowledge and the area in the world (developed vs. undeveloped)?	Thank you for your comment. Explanation regarding the relationship between knowledge and stigma and the are in the world have been added in discussion (lines 366-375).
